# Spinal Cord Termination and Lumbar Puncture Safety in Spinal Deformities

**DOI:** 10.7759/cureus.54820

**Published:** 2024-02-24

**Authors:** Abdullah S Alamri, Saleh A Almaktoum, Hamad A Alghanim, Ibrahim A Alqahtani, Jafar J Altammar, Mohammed S Alqahtani, Abdullah A Aldakheel, Rayan M Abdulhaq, Fahad A Aldawsari

**Affiliations:** 1 Neurology and Critical Care, King Fahad University Hospital, Al Khobar, SAU; 2 Neurology, Imam Abdulrahman Bin Faisal University, Dammam, SAU; 3 Neurology, King Fahad University Hospital, Al Khobar, SAU

**Keywords:** neuroimaging, termination of spinal cord, kyphoscoliosis, scoliosis, kyphosis, spinal deformity, lumbar puncture (lp)

## Abstract

Background

Lumbar puncture, a common diagnostic and therapeutic procedure, is performed regardless of individual spinal alignment variations. However, the impact of kyphosis, scoliosis, and kyphoscoliosis on spinal cord termination level and lumbar puncture safety remains unclear.

Objectives

This study aimed to determine if the termination level of the spinal cord is different in individuals with spinal deformities and to assess the necessity of routine neuroimaging for safe lumbar puncture localization.

Study design and settings

This single-center retrospective study was conducted at a university hospital using patients' electronic medical records. The study was focused on patients diagnosed with kyphosis, scoliosis, or kyphoscoliosis using spinal magnetic resonance imaging from January 2010 to December 2022.

Participants

We evaluated 240 patients: 120 with diagnosed spinal deformities (kyphosis, scoliosis, or kyphoscoliosis) and 120 without deformities, categorized by sex (deformed: 92 females, 28 males; non-deformed: 72 females, 48 males). Patients with spinal trauma, bleeding, or tumors were excluded.

Results

No statistically significant correlation was found between spinal deformities and spinal cord termination, with L1 remaining the most common endpoint in all groups.

Conclusion

Routine neuroimaging prior to lumbar puncture in patients with spinal deformities was not associated with a safer procedure due to no observed impact on the termination level of the spinal cord.

## Introduction

The vertebral column forms the central axis of the body. It consists of 33 vertebrae, including seven cervical, 12 thoracic, five lumbar, five sacral, and four coccygeal segments, in the cephalocaudal direction [1].

The spinal cord is a long, cylindrical, well-organized structure made up of nervous tissue composed of white and gray matter that extends from the foramen magnum as a continuation of the medulla oblongata in the brainstem down to the lower border of the first lumbar vertebra (L1) [2,3]. The spinal cord is located within the vertebral canal and is enclosed by three layers: the dura, arachnoid, and pia mater, which protect the spinal cord against damage [4]. The spinal cord can be divided into five regions: cervical, thoracic, lumbar, sacral, and coccygeal. It is divided into 31 segments consisting of 31 pairs of spinal nerves that contain motor, sensory, and autonomic fibers [3].

In adult males, the average spinal cord length is 45 cm, whereas it is 43 cm in females [5]. In terms of vertebral height, it has been observed that males have greater vertebral height than females. This could be attributed to the fact that the vertebrae of females are proportionally smaller than those of males [6]. At week eight of development, the spinal cord extends to the entire length of the vertebral canal. At birth, the conus medullaris terminated at the L2 level. In adults, the average termination level of the spinal cord is L1 in both males and females [7,8].

Kyphosis is the abnormally exaggerated outward curvature of the thoracic spine. In younger populations, normal kyphosis angles range between 20° and 40°; however, in older populations, the angle ranges from 48° to 50° in women and approximately 44° in men. The degree of kyphosis increases with age, with a higher rate in females [8,9].

Scoliosis is characterized by an abnormal lateral curvature of the spine. The normal degree of lateral curvature of the spine is <10°. The severity of scoliosis is determined by measuring the Cobb angle, which is the angle between two lines formed by drawing a perpendicular line between the upper endplate of the uppermost tilted vertebra and the lower endplate of the lowermost tilted vertebra. Scoliosis is considered when the Cobb angle is >10°. It is classified as mild (Cobb angle 10-20°), moderate (Cobb angle 20-40°), or severe (Cobb angle >40°) [10].

Kyphoscoliosis is a combination of kyphosis and scoliosis, an abnormal curvature of the vertebral column in both the sagittal and coronal planes, and may also involve rotation of the spinal axis. Kyphoscoliosis is predominantly observed in the thoracolumbar spinal region but can also occur in the cervicothoracic region. The degree and location of the abnormal curvature, the number of involved vertebrae, and the degree of axial rotation affect the curvature abnormality and clinical significance. Kyphoscoliosis can be classified as moderate or severe when the Cobb angle ranges from 25° to 100° or >100°, respectively [11].

Lumbar puncture is performed by inserting a thin needle at a point where a straight line connects the highest points of the iliac crests intersecting the vertebral column, which is visually recognized and confirmed by palpation and provides a guide to the fourth lumbar vertebral body. However, this line can cross the spine at a higher level between L1 and L2 and L4 and L5 and usually indicates a greater spinal level in females and obese patients [12,13]. Lumbar puncture is a relatively safe procedure; however, similar to any other procedure, complications may arise. Examples of complications include infection, cerebral herniation, bleeding, radicular pain, and numbness [14,15]. People with no abnormalities in their vertebral column are at risk of these complications, which raises the question of how people with potential variations in the spine, such as kyphosis and scoliosis, are affected by spinal procedures, such as lumbar puncture.

This study was conducted to determine if there is a need for certain neuroimaging measures (MRI) prior to lumbar puncture needle injection to potentially avoid any harm related to improper needle injection sites in individuals who have spinal deformities.

## Materials and methods

The study was approved by the Standing Committee for Research Ethics on Living Creatures at Imam Abdulrahman Bin Faisal University with an Institutional Review Board (approval number: IRB-UGS-2022-01-484, approval date: 21/11/2022). The procedures followed were in accordance with the ethical standards of the responsible committee on human experimentation (institutional or regional) and with the Helsinki Declaration of 1975, as revised in 2000.

This study examined the termination level of the spinal cord in adults with and without abnormal spinal curvatures using MRI scans. The research took place from September 25, 2022, to May 3, 2023. It analyzed retrospective data from the electronic medical records of patients who had spinal MRI scans from January 2010 to December 2022. The collected information included patient demographics, clinical data, and findings from the spinal MRI scans. All spine MRIs of patients diagnosed with kyphosis, scoliosis, or kyphoscoliosis and MRIs of patients without pathological vertebral curvatures were included. Patients who had trauma, bleeding, or tumors related to the spine were excluded. Finally, a total of 240 patients were included. There were 120 patients with non-deformities and 120 patients with spine deformities. The sampling technique involved creating a list of patients diagnosed with spinal deformities and having an MRI of the spine. From this list, a random selection of 120 patients was made, including patients with and without pathological vertebral curvatures.

Patients’ data and MRI images were accessed through the KFHU QuadraMed system (QuadraMed Corporation, Reston, Virginia, USA). Centricity software (GE HealthCare, Chicago, Illinois, USA) was used to measure the termination level of the spinal cord. Microsoft Excel (2019 Version, Microsoft Corporation, Redmond, Washington, USA) was used for data collection.

The data were collected in an Excel sheet that was divided between the researchers and organized into one sheet. Subsequently, tables were used to display the results. Statistical tests were performed using the Student’s t-test, analysis of variance (ANOVA), and chi-squared test. The results were analyzed using SPSS Statistics version 25 (IBM Corp. Released 2017. IBM SPSS Statistics for Windows, Version 25.0. Armonk, NY: IBM Corp.). Statistical significance was set at P<0.05.

The termination level of the spinal cord MRI scans underwent initial interpretation by seven independent final-year medical students under supervision by a neurology consultant, with subsequent revisions provided by the radiology department faculty at a university hospital. Moreover, the X-rays of the spine were reviewed, and the angle of deviation (Cobb angle) was measured.

## Results

Our cohort included 120 patients with deformities and 120 patients without deformities. In the deformed cases, 92 (76.7%) were females, and 28 (23.3%) were males, whereas in the non-deformed cases, 72 (60%) were females, and 48 (40%) were males. In the deformed cases, the age distribution showed 23 patients (19.2%) belonged to ≤18 years, 83 patients (69.2%) to 19-40 years, five patients (4.2%) to 41-60 years, and nine patients (7.5%) to ≥61 years (Table 1).

**Table 1:** Distribution of cases based on sex and age of patients

Deformity status	Age	Number/percentage	Female	Male	Total	p-value
Non-deformity	≤18	N	0	0	0	0.634
		%	0.0%	0.0%	0.0%	
	19-40	N	3	4	7	
		%	42.9%	57.1%	5.8%	
	41-60	N	27	17	44	
		%	61.4%	38.6%	36.7%	
	≥61	N	42	27	69	
		%	60.9%	39.1%	57.5%	
	Total	N	72	48	120	
		%	60.0%	40.0%	100.0%	
Deformity	≤18	N	14	9	23	0.164
		%	60.9%	39.1%	19.2%	
	19-40	N	66	17	83	
		%	79.5%	20.5%	69.2%	
	41-60	N	5	0	5	
		%	100.0%	0.0%	4.2%	
	≥61	N	7	2	9	
		%	77.8%	22.2%	7.5%	
	Total	N	92	28	120	
		%	76.7%	23.3%	100.0%	

The distribution of deformities showed that 114 (95%) patients had scoliosis, seven (5.8%) had kyphosis, and six (5%) had kyphoscoliosis. When we compared the distribution of deformities based on sex, females showed the majority of deformities, where scoliosis was seen in 88 (73.3%), kyphosis in six (85.7%), and kyphoscoliosis in five (83.3%) of patients. When we compared the distribution of deformities based on age groups, the majority of the cases of scoliosis were seen in the 19-40 year age group, representing 80 (70.2%) patients, whereas kyphosis and kyphoscoliosis were seen more in the ≤18 year age group, accounting for three (42.9%) and three (50%) of the cases, respectively (Table 2).

**Table 2:** Distribution of deformities based on age and sex

Deformity type	Scoliosis						Kyphosis						Kyphoscoliosis					
Sex	Female		Male		Total		Female		Male		Total		Female		Male		Total	
Number/percentage	N	%	N	%	N	%	N	%	N	%	N	%	N	%	N	%	N	%
≤18 years	14	15.9	8	30.8	22	19.3	3	50	0	0	3	42.9	3	60	0	0	3	50
19-40 years	64	72.7	16	61.5	80	70.2	1	16.7	1	100	2	28.6	1	20	1	100	2	33.3
41-60 years	4	4.5	0	0	4	3.5	0	0	0	0	0	0	0	0	0	0	0	0
≥61 years	6	6.8	2	7.7	8	7	2	33.3	0	0	2	28.6	1	20	0	0	1	16.7
Total	88	73.3	26	21.7	114	100	6	85.7	1	14.3	7	100	5	83.3	1	16.7	6	100

The distribution at the end of the spinal cord showed that L1 was the most common endpoint in 173 (72.1%) cases, followed by T12 in 38 cases (15.8%), and L2 in 23 cases (9.8%). However, there were no statistically significant differences in the distribution of the ends of the spinal cord between the deformed and non-deformed patients (P=0.255) (Table 3).

**Table 3:** Comparison of the end of the spinal cord between deformed and non-deformed patients

			Type		Total	p-value for chi-squared test
			Non-deformity	Deformity		
End of spinal cord	T11	N	0	1	1	0.255
		%	0.0%	100.0%	0.4%	
	T11–T12	N	0	1	1	
		%	0.0%	100.0%	0.4%	
	T12	N	23	15	38	
		%	60.5%	39.5%	15.8%	
	L1	N	85	88	173	
		%	49.1%	50.9%	72.1%	
	L1–L2	N	0	3	3	
		%	0.0%	100.0%	1.3%	
	L2	N	12	11	23	
		%	52.2%	47.8%	9.6%	
	L4	N	0	1	1	
		%	0.0%	100.0%	0.4%	

The mean angle of deviation was significantly higher in deformed patients (36.70 ± 21.1) when compared to non-deformed patients (4.010 ± 3.2, P<0.001). Among deformed patients, the mean angle of deviation was found to be lesser in patients that end at the level of L1 of the spinal cord (32.80 ± 17.2) when compared to other cases (P<0.001). The mean age of the deformed patients was significantly less (27.0 ±15.0 years) than that of the non-deformed patients (62.2 ±12.6, P<0.001) (Table 4).

**Table 4:** Comparison of age and angle of deviation based on the end of the spinal cord

Parameters	Deformity status	End of spinal cord	N	Mean	Standard deviation	p-value	
Age	Non-deformed	T12	23	61.7	12.1	0.674	<0.001
		L1	85	61.8	12.9		
		L12	12	66.1	12.3		
		Total	120	62.2	12.6		
	Deformed	T11	1	14	-	<0.001	
		T11-T12	1	20	-		
		T12	15	32.7	18.7		
		L1	88	27.2	15.1		
		L1-L2	3	16.3	12.7		
		L2	11	23.3	7.9		
		L4	1	21	-		
		Total	120	27	15		
Angle of deviation	Non-deformed	T12	23	3.5	2.4	0.538	<0.001
		L1	85	4.2	3.4		
		L2	12	3.7	3.6		
		Total	120	4.01	3.2		
	Deformed	T11	1	32.5	-	0.492	
		T11-T12	1	115	-		
		T12	12	37.9	23.1		
		L1	78	32.8	17.2		
		L1-L2	2	81	2.8		
		L2	7	44.3	25.4		
		L4	1	66	-		
		Total	120	36.7	21.1		

When we compared the cases who underwent MRI pre- and post-surgical correction in the deformed patients, the majority of cases had an endpoint of the spinal cord at the level of L1 preoperatively. Specifically, L1 was identified as the pre-operative endpoint in eight cases, while L2 was reported as the pre-operative endpoint in two cases. Post-operatively, six patients had L1, three patients had L1-L2, and one patient had T12-L1 (Table 5).

**Table 5:** Comparison of the end of the spinal cord between pre- and post-operative correction in deformed patients

		End of spinal cord (pre-operative correction)		Number/percentage	p-value
		L1	L2	Total	0.732
End of spinal cord (post-operative correction)	T12-L1	1	0	1	
		100.00%	0.00%	100.00%	
	L1-L2	2	1	3	
		66.70%	33.30%	100.00%	
	L1	5	1	6	
		83.30%	16.70%	100.00%	

While comparing the end of the spinal cord in all deformed patients who had surgery with or without MRI and patients who didn't undergo surgery, it can be observed that the most common endpoint of the spinal cord was at the level of L1. Among these patients, 62 cases (70.5%) had no surgery, 18 cases (20.5%) didn't undergo MRI post-operative correction, and eight (9.1%) cases with MRI showed variations at the endpoint of the spinal cord. Additionally, the second most common endpoint was T12, accounting for 13 cases (86.7%) that didn't undergo surgical correction, while two cases (13.3%) had no MRI post-operative correction. Other endpoints such as T11, T11-T12, L1-L2, L2, and L4 were also observed, although with lower frequencies (Table 6).

**Table 6:** Comparison of the end of the spinal cord between pre- and post-operative correction in all deformed patients

			Post-operative correction				
		Number/percentage	No surgery	No MRI after surgery	L1	L1-L2	T12–L1
Pre-operative correction	T11	N	0	1	0	0	0
		%	0.0%	100.0%	0.0%	0.0%	0.0%
	T11-T12	N	1	0	0	0	0
		%	100.0%	0.0%	0.0%	0.0%	0.0%
	T12	N	13	2	0	0	0
		%	86.7%	13.3%	0.0%	0.0%	0.0%
	L1	N	62	18	5	2	1
		%	70.5%	20.5%	5.7%	2.3%	1.1%
	L1-L2	N	3	0	0	0	0
		%	100.0%	0.0%	0.0%	0.0%	0.0%
	L2	N	8	1	1	1	0
		%	72.7%	9.1%	9.1%	9.1%	0.0%
	L4	N	1	0	0	0	0
		%	100.0%	0.0%	0.0%	0.0%	0.0%

After surgical correction, a change at the end of the spinal cord was observed in five patients (50%). In five cases, the endpoint remained at L1, indicating no change from the pre-operative state. Two cases demonstrated a transition from L1 to L1-L2 at the endpoint of the spinal cord. Additionally, one case exhibited a transition from L1 to T12-L1. Furthermore, there was a shift from L2 to L1 in one case, and another case demonstrated a shift from L2 to L1-L2 (Table 7).

**Table 7:** Comparison of change of the end of the spinal cord in cases with MRI done after surgery (N=10)

End of spinal cord (pre-operative)	End of spinal cord (post-operative)	Change of end of spinal cord
L1	T12-L1	Yes
L1	L1	No
L1	L1	No
L2	L1	Yes
L1	L1	No
L1	L1	No
L1	L1	No
L1	L1-L2	Yes
L2	L1-L2	Yes
L1	L1-L2	Yes

## Discussion

Study results showed that neuroimaging studies may not be needed as a precautionary measure for patients exhibiting vertebral column variation who undergo spinal procedures such as lumbar puncture, as there is no remarkable statistical correlation between the variations in the spine (kyphosis, scoliosis, and kyphoscoliosis) and the spinal cord endpoint. Our study showed that L1 was the most common termination point (Table 3), which notably is the most common termination level, as reported by Mbaba et al. and Moon et al. [16,17]. Mbaba et al. conducted a study with a sample size of 177 patients and found that the majority of the spinal cord was terminated at the L1 level [16]. Furthermore, Moon et al. found that the most common end level of the spinal cord was L1 in 189 Korean patients [17].

Our findings showed that the angle of deviation was significantly increased in patients with spinal deformities compared with non-deformed patients, with the most common spinal deformities in our study being scoliosis, kyphosis, and kyphoscoliosis, respectively (Tables 2, 4) [10].

In our study, we noticed that females had a higher chance of being associated with spine deformities than their male counterparts (Table 1). Fon et al. found that the degree of kyphosis increases faster in females than males [8]. Furthermore, scoliosis is more common among females than males, with a 3:1 ratio [18]. In contrast, another study showed no sex-based difference in spinal deformities [19]. Moreover, the most common age group in our data was between 19 and 40 years of age, followed by patients aged 18 and younger, patients aged 61 and older, and finally patients aged 41 and 60 years (Table 2). Other studies have shown a correlation between an increase in age and kyphosis as a direct result of the progressive decline in various types of structures, such as bones, muscles, ligaments, and tendons [8]. Additionally, aging increases the likelihood of developing scoliosis, which can be attributed to progressive degenerative diseases such as facet and progressive disc degeneration [20].

Our findings revealed that the implementation of spinal correction surgery in individuals presenting with spinal deformities resulted in a notable change in the termination level of the spinal cord in 50% of the patients (Tables 5-7). This is supported by Hong et al.'s study, which states that spinal corrective surgery affects the end of the spinal cord in patients with pathological spinal curvatures [21].

This study has some limitations. With the limited number of patients (n=240) and the uneven distribution of sex and age groups, e.g., spine-deformed patients were distributed as 92 (76.7%) and 28 (23.3%) females and males, respectively, in addition to the spine-non-deformed patients being 72 (60%) and 48 (40%) females and males, respectively. Regarding the age distribution of deformed cases, the prevalence was highest among individuals aged 19-40, accounting for 83 cases (69.2%). Subsequently, the age group of 18 years and younger constituted 23 cases (19.2%), while individuals aged 61 years and older accounted for nine cases (7.5%), and the age group of 41-60 years represented five cases (4.2%). Furthermore, interpretations of radiological images, such as MRI, may exhibit subjectivity, thereby rendering them susceptible to human error. We recommend increasing the sample size to improve the accuracy and applicability of this study.

## Conclusions

Our research assessed the necessity of conducting spinal MRI scans before lumbar punctures in patients with spinal deformities to avoid incorrect needle placement. Results revealed that spinal variations such as kyphosis, scoliosis, or kyphoscoliosis had no significant impact on the spinal cord endpoint, which commonly occurs at L1. Therefore, the routine use of spinal MRI for lumbar puncture preparation in those with spinal deformities seems unwarranted, which could simplify the pre-procedural assessment and decrease the use of healthcare resources.
